# The cardiac conundrum: a systematic review and bibliometric analysis of authorship in cardiac magnetic resonance imaging studies

**DOI:** 10.1186/s13244-020-00850-1

**Published:** 2020-02-27

**Authors:** Renato Cuocolo, Andrea Ponsiglione, Serena Dell’Aversana, Ludovica D’Acierno, Giulia Lassandro, Lorenzo Ugga, Valeria Romeo, Elena Augusta Vola, Arnaldo Stanzione, Francesco Verde, Valentina Picariello, Iolanda Capaldo, Giuseppe Pontillo, Valeria Cantoni, Roberta Green, Mario Petretta, Alberto Cuocolo, Massimo Imbriaco

**Affiliations:** 1 Department of Advanced Biomedical Sciences, University Federico II, Sergio Pansini 5, 80138 Naples, Italy; 2 Department of Translational Medical Sciences, University Federico II, Naples, Italy

**Keywords:** Magnetic resonance imaging, Systematic review, Heart

## Abstract

**Purpose:**

We aimed to assess the role of radiologists, cardiologists, and other medical and non-medical figures in cardiac magnetic resonance imaging (MRI) research in the last 34 years, focusing on first and last authorship, number of published studies, and journal impact factors (IF).

**Methods:**

Articles in the field of cardiac MRI were considered in this systematic review and retrospective bibliometric analysis. For included studies, the first and last authors were categorized as cardiologists, radiologists/nuclear medicine physicians, medical doctors (MD) with specialties in both cardiology and radiology/nuclear medicine, and other MD and non-MD. Differences in the number of papers published overall and by year and institution location for the first and last author category were assessed. Mean IF differences between author categories were also investigated.

**Results:**

A total of 2053 articles were included in the final analysis. For the first authors (*n* = 2011), 52% were cardiologists, 22% radiologists/nuclear medicine physicians, 16% other MD, 10% other non-MD, and 1% both cardiologists and radiologists/nuclear medicine physicians. Similarly, the last authors (*n* = 2029) resulted 54% cardiologists, 22% radiologists/nuclear medicine physicians, 15% other MD, 8% other non-MD, and 2% both cardiologists and radiologists/nuclear medicine physicians. No significant differences due to institution location in the first and last authorship proportions were found. Average journal IF was significantly higher for cardiologist first and last authors when compared to that of radiologists/nuclear medicine physicians (both *p* < 0.0001).

**Conclusion:**

Over 50% of studies in the field of cardiac MRI published in the last 34 years are conducted by cardiologists.

## Key points

- Fifty-two percent of first authors were cardiologists followed by 22% medical imaging physicians.
- Fifty-four percent of last authors were cardiologists followed by 22% medical imaging physicians.
- Institution location was not a significant factor for author category distribution.
- Mean journal impact factor was significantly higher for cardiologist first and last authors.

## Introduction

The importance of cardiovascular (CV) imaging has greatly increased over the years. Developments in non-invasive techniques such as echocardiography, computed tomography, and magnetic resonance imaging (MRI) have contributed to this trend [1]. This is also reflected in the scientific literature, with more and more papers being published employing CV imaging [2]. Interest in and use of cardiac MRI have also increased as it allows for morphological and functional imaging of the heart, with a wide range of indications [3–9]. Yet, there is some overlap in both clinical practice and research in regard to the role of different medical specialties, especially radiology and cardiology, leading to some debate [10]. Several articles have been published on this topic, ranging from editorials to literature reviews [2, 11–13]. In the past, the role of these two specialties has been found to be essentially equal, with a balanced output in the scientific literature in the field of CV imaging overall and cardiac MRI [12]. Since the last comprehensive assessment, the use of this imaging procedure has spread and it has been validated for more applications, with studies conducted on real-world patients increasing compared to preliminary investigations on animal, phantom, and healthy volunteer populations. This has led to the need for a new assessment of the current state of cardiac MRI literature and publication trends over the years.

To assess the role of radiologists, cardiologists, and other medical and non-medical figures in cardiac MRI research over the years, we performed a systematic review and bibliometric analysis of the literature focusing on first and last authorship, number of papers, and journal impact factors (IF).

## Methods

This systematic review and bibliometric analysis were conducted by assessing all studies in the English language available on the National Library of Medicine Medline and Web of Science databases up to September 30, 2018. The search string included the following: “Cardiac Magnetic Resonance Imaging”[Title] OR “Cardiac Magnetic Resonance”[Title] OR “Cardiac MRI”[Title] OR “Cardiac MR”[Title] OR “CMRI”[Title] OR “CMR”[Title]. After the removal of duplicates, titles and abstracts were screened to remove articles not on cardiac MRI, case reports, letters, editorials, reviews, systematic reviews, meta-analyses, guidelines, position papers, and studies not on a human population (i.e., animal models, public datasets, methodological, or ex vivo).

For included studies, the first and last authors were categorized as cardiologists, radiologists/nuclear medicine physicians (medical imaging), medical doctors (MD) with specialties in both cardiology and radiology/nuclear medicine, and other MD and non-MD. In detail, author’s background was obtained from publicly available information, such as online curriculum vitae and profiles on social networks (e.g., LinkedIn, ResearchGate). When an attribution was not obtainable, the author was excluded from further analysis. Residents were classified in the corresponding specialty group and students as other MD. In case of shared authorship, only the first and last author’s background was analyzed. Author institution location was noted and categorized as belonging to one of the following areas for subsequent analysis: “Europe,” “North America,” “Far East,” and “Rest of the World.” Furthermore, journal and year of publication were recorded in order to obtain the corresponding IF.

### Statistical analysis

A statistical analysis was conducted to assess differences in the number of articles published overall and by year for the first and last author category. Similarly, differences in mean IF for the first and last author category were analyzed. Furthermore, the pairings of the first and last author categories were assessed. Finally, as previous studies showed an equal proportion of publications between cardiologists and radiologists, proportion equivalence tests were conducted on papers published only by these authors to assess if they were significantly different both in our observation and in relation to expected results from past studies. Pearson’s *χ*^2^ test was used to assess differences in author category proportions across different institution locations. A *p* value < 0.05 was considered statistically significant. All statistical analyses were performed on the Stata software (StataCorp 2015, Stata Statistical Software: Release 14, College Station, TX: StataCorp LP).

## Results

**Fig. 1:**
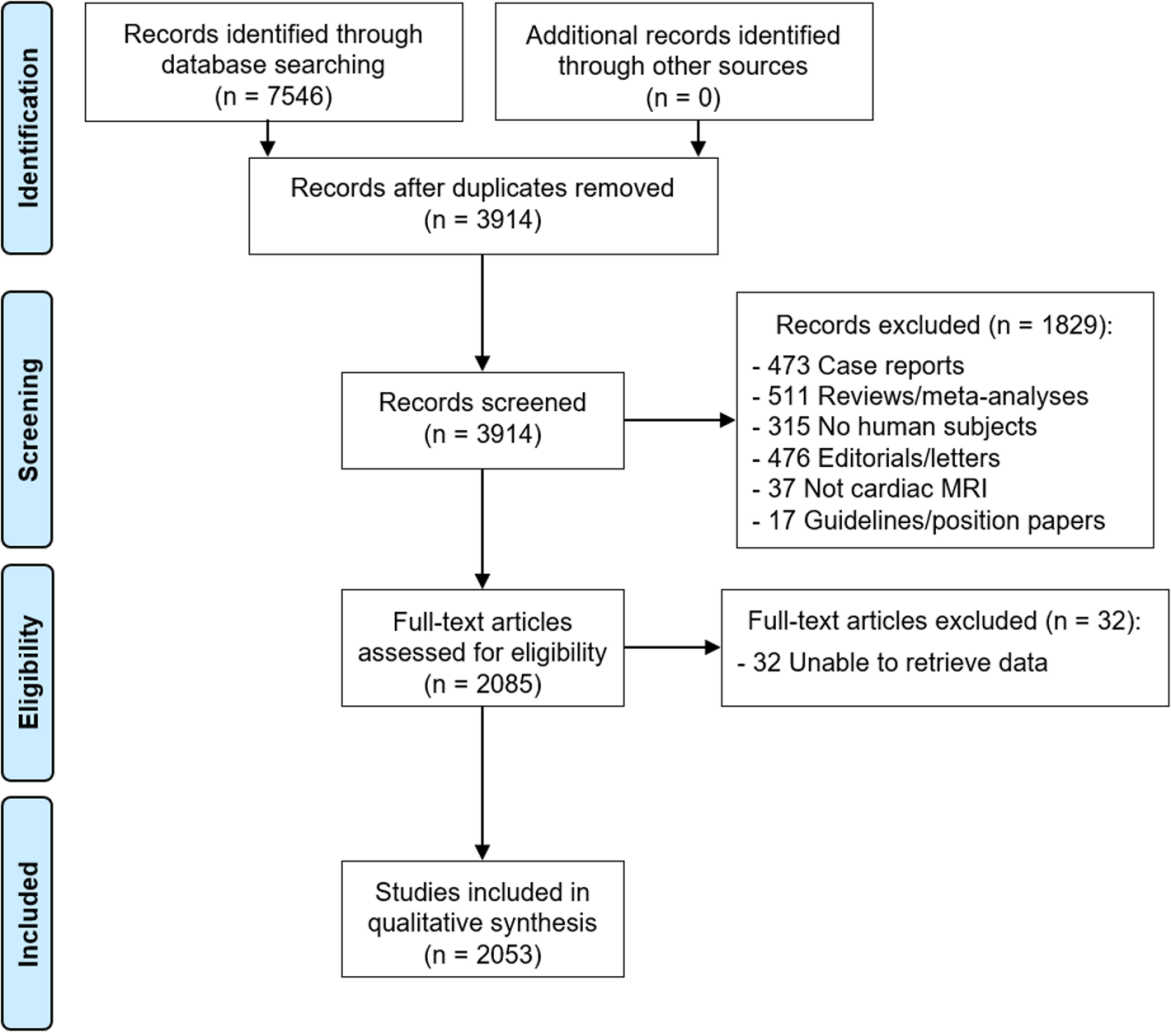
Literature review and study selection process flowchart

The study selection process is shown in Fig. 1. From the initial literature search, 7546 articles were found. After removal of duplicates and application of exclusion criteria, 2085 studies were analyzed for data collection. In 32 cases, no information was obtainable for both first and last authors, leading to the exclusion of the paper. Therefore, a total of 2053 studies were included in the qualitative synthesis. The first author information was retrievable for 2011 papers, and the last author for 2029.

For the first authors, 1036 (52%) were cardiologists, 433 (22%) radiologists/nuclear medicine physicians, 318 (16%) other MD, 196 (10%) other non-MD, and 28 (1%) both cardiologists and radiologists/nuclear medicine physicians. Similarly, 1087 (54%) last authors were cardiologists, 444 (22%) radiologists/nuclear medicine physicians, 310 (15%) other MD, 153 (8%) other non-MD, and 35 (2%) both cardiologists and radiologists/nuclear medicine physicians.

The results of the proportion analysis of papers only published by cardiologists and radiologists/nuclear medicine physicians as the first or last authors are reported in Table 1. For the first authorship, cardiologists show a significantly higher presence both overall (*n* = 1036; 71%; *p* < 0.0001) and for all sub-category assessments (proportion range = 69–72%; *p* < 0.0001). A similar situation was observed for the last authorship with 70% of papers (*n* = 1019) having a cardiology specialist, with similar distributions in all categories (proportion range = 69–70%; all *p* < 0.0001). Finally, our findings were also significantly different (*p* < 0.0001 for both first and last authors) when compared to the expected proportions (50% each).

**Table 1:** Overall authorship proportions by study sub-categories for articles published by cardiologists or radiologists/nuclear medicine physicians as the first or last authors

	Cardiology	Radiology/nuclear medicine	*P* value
First author			
All studies	71% (68–73%)	29% (27–32%)	< 0.0001
Retrospective studies	69% (65–73%)	31% (27–35%)	< 0.0001
Prospective studies	72% (69–75%)	28% (26–31%)	< 0.0001
Pediatric population	70% (62–78%)	30% (22–38%)	< 0.0001
Adult population	70% (68–73%)	30% (27–32%)	< 0.0001
Last author			
All studies	70% (67–72%)	30% (29–33%)	< 0.0001
Retrospective studies	69% (65–73%)	31% (27–35%)	< 0.0001
Prospective studies	70% (67–73%)	30% (27–33%)	< 0.0001
Pediatric population	70% (62–79%)	30% (21–38%)	< 0.0001
Adult population	69% (67–72%)	31% (28–33%)	< 0.0001

Values are expressed as proportions (95% confidence interval)

**Fig. 2:**
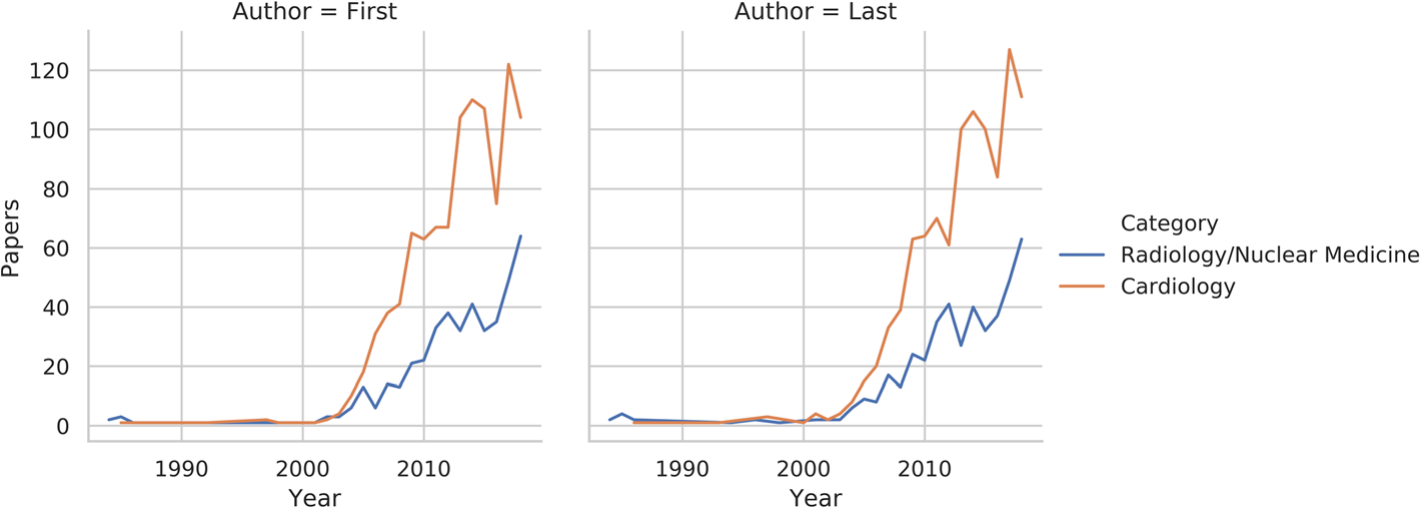
Line plot depicting the number of studies published by cardiologists or radiologists/nuclear medicine physicians as the first or last authors over the years

**Fig. 3:**
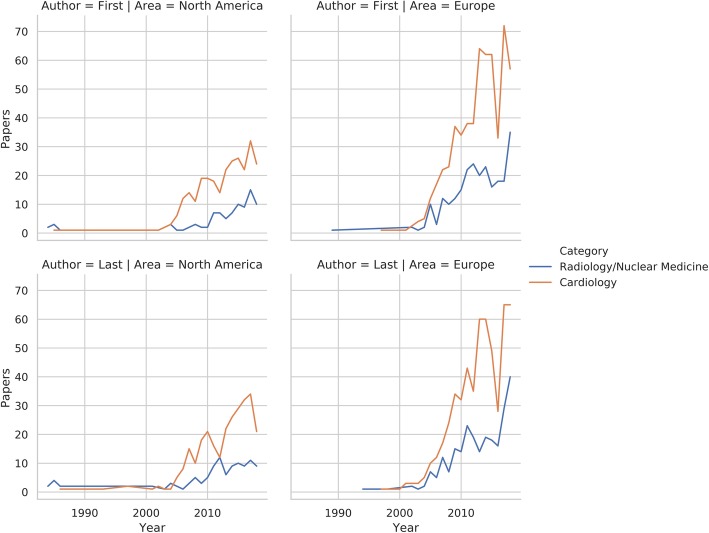
Line plot depicting the number of studies published by cardiologists or radiologists/nuclear medicine physicians as the first or last authors over the years in Europe and North America

The distribution of papers in relation to publication year and first/last author category is shown in Fig. 2. Regarding first author locations, 1073 (53%) of papers were published by European institutions, 582 (29%) in North American ones, 227 (11%) in the Far East, and 129 (7%) in the rest of the world. Similarly, the respective values for last authors were 1060 (52%), 607 (30%), 230 (11%), and 132 (7%). The institution geographical analysis was limited to the two most frequent first/last author categories and locations, i.e., cardiologists and radiologists based in Europe and North America (Table 2). No significant differences were found in terms of author category proportions for both first (*p* = 0.14) and last (*p* = 0.28) authors in either location. Figure 3 depicts the distribution of papers in relation to publication year and author category within each of the institution locations included in the analysis. The data regarding the numerosity of all author categories in each of the institution location categories is reported in the supplementary materials.

**Table 2:** Authorship by institution location for articles published by cardiologists or radiologists/nuclear medicine physicians as the first or last authors

	Cardiology	Radiology/nuclear medicine	Total
First author			
Europe	584 (71%)	244 (29%)	828 (69%)
North America	272 (75%)	92 (25%)	364 (31%)
Total	856 (72%)	336 (28%)	
Last author			
Europe	547 (69%)	245 (31%)	792 (67%)
North America	280 (72%)	108 (28%)	388 (33%)
Total	827 (70%)	353 (30%)	

Values are expressed as absolute numbers (percentage within each geographical area or of the total number of papers)

**Fig. 4:**
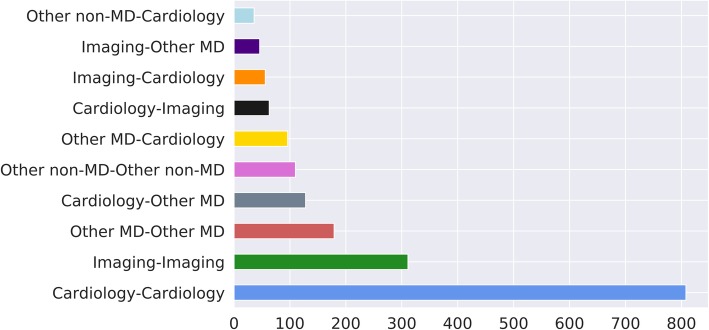
Bar plot showing the total number of studies grouped by the ten most frequent first and last author category pairings

With regard to the first and last author category pairings, among the 1998 papers for which both author categories were recorded, the most common were cardiology-cardiology (*n* = 808; 40%), followed by radiology/nuclear medicine-radiology/nuclear medicine (*n* = 311; 16%), other MD-other MD (*n* = 179; 9%), cardiology-other MD (*n* = 128; 6%), and other non-MD-other non-MD (*n* = 110; 6%) (Fig. 4).

The average IF was significantly (*p* < 0.0001) higher for cardiologist first (IF = 4.59 ± 3.85) and last (IF = 4.17 ± 3.28) authors when compared to that of radiologists/nuclear medicine physicians (IF = 3.43 ± 2.94 and 3.39 ± 2.58, respectively). For the first authors, cardiologist mean IF was also higher when compared to all other author categories (other-MD IF = 4.04 ± 3.4, *p =* 0.03; other non-MD IF = 3.43 ± 2.20, *p* < 0.0001), while it was only higher than other non-MDs for the last authors (other-MD IF = 4.10 ± 3.51, *p =* 0.759; other non-MD IF = 3.31 ± 1.78, *p* = 0.003). As for radiologists/nuclear medicine physicians, it was significantly higher for both first (*p* = 0.014) and last (*p* = 0.002) authorship only when compared to other-MDs.

## Discussion

Radiologists and nuclear medicine physicians have always been associated with advances and innovations in CV imaging and introduction of new techniques for the clinical management of patients with heart diseases [14–16]. Although numerous CV imaging modalities, such as echocardiography, angiocardiography, and coronary angiography, have been developed by radiologists, there was a progressive reduction of the role of radiologists in this field as these have been increasingly or exclusively performed by cardiologists [17, 18]. Reasons for this include the fact that they are charged with clinical management of the patients, while radiologists rarely have direct access or interactions with patients.

The increasing use and application of cardiac MRI has led to a revitalization of the radiologists’ role in CV imaging [13]. Cardiac MRI is a widely accepted advanced cross-sectional imaging modality for functional and anatomical evaluation as well as tissue characterization of several CV disorders [19–21]. The last 20 years has seen an explosion of research in cardiac-MRI, and the potential of this technique to accurately depict in one single section, myocardial morphology, function, and perfusion, has led it to become a powerful tool in the diagnostic work up of patients with CV diseases [22–25]. In particular, this technique is mainly used in clinical practice for the assessment of congenital heart disease, ischemic and non-ischemic cardiomyopathies, valvular and pericardial diseases, and myocardial tumor [3, 16, 25–29]. Consequently, there has been a steady increase and a growing trend of scientific publications pertaining to the field of cardiac MRI.

The constant increase in clinical use of cardiac MRI over the decades has led to a real conflict between radiologists and cardiologists over who should be performing and interpreting these procedures [6, 17]. In parallel with the expanded number of MRI scans performed, the volume of scientific production has been continuously increasing [10, 11, 30]. Many different professional expertise has contributed to enrich scientific literature with an ever-growing number of papers involving cardiac MRI, ranging from editorials to literature reviews [31]. The aim of this study, as already done in other fields [32], was to get deeper into the role played by MD, especially radiologists/nuclear medicine physicians and cardiologists, and non-MD, in clinical cardiac MRI studies, analyzing the original researches published on this topic in the last 34 years.

Dasit et al. retrospectively evaluated the number of articles on cardiac MRI written by authors from radiology and cardiology departments between 1999 and 2004 and showed that the number of cardiac MRI studies by radiologists (49.5%) and by cardiologists (50.5%) was balanced [12]. These authors also found that radiologists published more articles on developing techniques and used animals and combinations of volunteers and technical material more often in their studies than did cardiologists. Conversely, results of most clinical trials, controlled clinical trials, and randomized controlled trials were mostly published by cardiologists. In our search, the number of articles in the late 1990s and early 2000s is lower, probably due to different search methods. In contrast to those authors, we focused on prospective and retrospective clinical trials conducted on human patients. This explains the differences both in terms of papers found and in proportion of publication. Our findings are in accordance with theirs in relation to the dominance of cardiologists in this setting. It is also true that the technique has greatly matured over the years and achieved greater diffusion in clinical practice. Today, the vast majority of research articles are centered on clinical trials, and the development of new sequences or technical developments has passed mostly in the hands of engineers and other non-medical figures as they have become increasingly complex. This was the rationale that induced us to adapt the search criteria to the current setting and to focus on the use of cardiac MRI in relation to actual human patients, a topic we believed more appropriate in determining who assumed a leading role in cardiac MRI both in scientific production and clinical practice.

As can be seen in our results, with the growing production of papers in cardiac MRI, the disproportion between cardiologists and other figures, including medical imaging specialists, has increased. Overall, cardiologists represent over 50% of the first and last authors independently of the prospective or retrospective nature of the study or if the focus is on adult or pediatric patient populations. Geographical location did not significantly influence this trend. This is interesting as differences in legislation, exam reimbursement, and national health systems could have been expected to have an impact. It should also be noted that while European authors have started publishing later than their North American counterparts, they currently represent the main source of papers in cardiac MRI. However, these two areas together are still responsible for most of the scientific production in this area. Similarly, the pairing of cardiologists in both positions was clearly more common (40%) than any other. These differences are so overwhelming that it cannot be denied that cardiologists are currently the leading figure in cardiac MRI. This is also reflected by the fact that the most followed clinical practice guidelines, including indications and management of MRI procedures, are developed by cardiological scientific societies.

This development could also limit the benefits that could be obtained by a more prominent role of radiologists. In the ongoing debate over who should lead the field of cardiac MRI, radiologists usually advocate that they receive more formal training in MRI while cardiologists lack adequate experience and training using the technology [10]. The expertise of radiologists is also reflected in other aspects of CV imaging such as the assessment of extra-cardiac findings [33]. Conversely, cardiologists argue that they are the ones tasked with making clinical decisions and recommendations based on their qualification and training and consequently should be the leading actors in this field. It is also interesting to note that a minority of MDs solved the issue with the acquisition of a dual specialty in cardiology and medical imaging. While this in part can be due to other reasons (i.e., legal requirements for MRI reporting in some countries), it is also a sign of the added value that both trainings can bring to the field and a possible suggestion for a further evolution of CV imaging with a new, dedicated, and specific training program.

A possible argument of discussion is that what really matters for the best interest of patients is the added specific value that both radiologists and cardiologists can bring to CV imaging. Hopefully, in the future, both figures can work more closely together, sharing individual expertise and peculiarities. The establishment of a combined figure, a “cardiac imaging specialist” or a “cardio-radiologist,” would be helpful, with the ultimate goal of a better patient care. Otherwise, it is likely that collaboration between the two fields will continue to be a struggle.

## Study limitations

This paper presents some limitations that should be acknowledged. The first and last authors are only two members of a larger team. Their specialty does not give the whole picture of contributions to a scientific paper. However, the first and last authors are usually responsible for study design and overview, and their role is usually more relevant than that of other members. The exclusion of studies not employing human patients could have favored the dominance of cardiologists, but this better reflects the current clinical situation, and the result from a large number of studies was so overwhelming that there is little doubt it could have significantly changed.

## Supplementary information

**Additional file 1.** Supplementary Table 1. Authorship by institution location for all first and last author categories.

## Data Availability

Not applicable.

## Abbreviations

CV: Cardiovascular
IF: Impact factor
MD: Medical doctor
MRI: Magnetic resonance imaging
