# Supplementary material for: The cardiac conundrum: a systematic review and bibliometric analysis of authorship in cardiac magnetic resonance imaging studies
Source: Insights Imaging. 2020 Feb 27;11:42. doi: 10.1186/s13244-020-00850-1 (PMC7046856; doi:10.1186/s13244-020-00850-1)
Supplement: Supplementary file 1 — Additional file 1. Supplementary Table 1. Authorship by institution location for all first and last author categories. [file 13244_2020_850_MOESM1_ESM.doc]

**Supplementary Table 1.** Authorship by institution location for all first and last author categories.

|  | **Cardiology** | **Radiology/Nuclear medicine** | **Cardiology and Radiology/Nuclear medicine** | **Other MD** | **Other non-MD** | **Total** |
| --- | --- | --- | --- | --- | --- | --- |
| First author |  |  |  |  |  |  |
| Europe | 584 (54%) | 244 (23%) | 19 (2%) | 141 (13%) | 85 (8%) | 1073 (53%) |
| North America | 272 (47%) | 92 (16%) | 9 (2%) | 135 (23%) | 74 (13%) | 582 (29%) |
| Far East | 99 (43%) | 74 (33%) | 0 (0%) | 27 (12%) | 27 (12%) | 227 (11%) |
| Rest of the World | 81 (63%) | 23 (18%) | 0 (0%) | 15 (12%) | 10 (8%) | 129 (6%) |
| Last author |  |  |  |  |  |  |
| Europe | 547 (52%) | 245 (23%) | 17 (2%) | 181 (17%) | 70 (7%) | 1060 (52%) |
| North America | 280 (46%) | 108 (18%) | 13 (2%) | 146 (24%) | 60 (10%) | 607 (30%) |
| Far East | 110 (48%) | 70 (30%) | 1 (1%) | 36 (16%) | 13 (6%) | 230 (11%) |
| Rest of the World | 82 (62%) | 18 (14%) | 2 (2%) | 20 (15%) | 10 (8%) | 132 (7%) |

*Values are expressed as absolute numbers (percentage within each geographical area or of the total number of papers)*
